# The Use of Recombinant Pseudotype Virus-Like Particles Harbouring Inserted Target Antigen to Generate Antibodies against Cellular Marker p16^INK4A^

**DOI:** 10.1100/2012/263737

**Published:** 2012-04-26

**Authors:** Rita Lasickienė, Alma Gedvilaite, Milda Norkiene, Vaida Simanaviciene, Indre Sezaite, Dovile Dekaminaviciute, Evelina Shikova, Aurelija Zvirbliene

**Affiliations:** ^1^Institute of Biotechnology, Vilnius University, Graiciuno 8, 02241 Vilnius, Lithuania; ^2^Institute of Experimental Morphology, Pathology and Anthropology with Muzeum, Bulgarian Academy of Sciences, Acad. G. Bonchev Street, Building 25, 1113 Sofia, Bulgaria

## Abstract

Protein engineering provides an opportunity to generate new immunogens with desired features. Previously, we have demonstrated that hamster polyomavirus major capsid protein VP1-derived virus-like particles (VLPs) are highly immunogenic and can be employed for the insertion of foreign epitopes at certain surface-exposed positions. In the current study, we have designed pseudotype VLPs consisting of an intact VP1 protein and VP2 protein fused with the target antigen—cellular marker p16^INK4A^—at its N terminus. Both proteins coexpressed in yeast were self-assembled to pseudotype VLPs harbouring the inserted antigen on the surface. The pseudotype VLPs were used for generation of antibodies against p16^INK4A^ that represents a potential biomarker for cells transformed by high-risk human papillomavirus (HPV). The pseudotype VLPs induced in immunized mice a strong immune response against the target antigen. The antisera raised against pseudotype VLPs showed specific immunostaining of p16^INK4A^ protein in malignant cervical tissue. Spleen cells of the immunized mice were used to generate monoclonal antibodies against p16^INK4A^ protein. The specificity of antibodies was proven by the immunostaining of HPV-transformed cells. In conclusion, the current study demonstrates the potential of pseudotype VLPs with inserted target antigen as a new type of immunogens to generate antibodies of high diagnostic value.

## 1. Introduction

Gene and protein engineering provides an opportunity to generate novel chimeric proteins with desired features, such as enhanced immunogenicity. Structural proteins originating from human and animal viruses, for example, papilloma, hepatitis B, and parvo- and rotaviruses with their intrinsic capacity to self–assemble to highly organized structures—virus-like particles (VLPs)—have been shown to possess high immunogenicity and therefore exploited as potential vaccines [1–3]. Moreover, recombinant VLPs can be employed as carriers for non immunogenic proteins or peptides in order to enhance their immunogenicity. Previous studies demonstrated that insertions/fusions of foreign protein segments at certain sites of VLP carriers derived from papilloma-, polyoma-, hepadna-, parvo-, and retroviruses did not influence protein folding and assembly of chimeric VLPs. The immunogenicity of foreign sequences presented on the surface of chimeric VLPs is enhanced making these VLPs promising vaccine candidates [4–7]. Recently, we have demonstrated that hamster polyomavirus (HaPyV) major capsid protein VP1-derived VLPs are highly immunogenic and tolerate inserts of different size and origin at certain surface-exposed positions. The chimeric HaPyV-VP1 VLPs have been shown to activate efficiently the antigen-presenting cells and induce strong insert-specific B- and T-cell responses in mice [8, 9]. These studies demonstrated that chimeric VLPs represent promising novel immunogens to generate monoclonal antibodies (MAbs) of the desired epitope-specificity. The main advantage of chimeric VLPs over tradicional immunogens such as synthetic peptides chemically coupled to carrier proteins is the exposure of the target sequence on the surface of VLPs thus allowing its accessibility to the B cells [9]. Although chimeric VLPs tolerate inserts up to 120 amino acid (aa) residues, the insertion of longer protein sequences generally affects proper folding and self-assembly of VLPs (our unpublished observation). Therefore, new approaches for enhancing the immunogenicity of long protein segments or full-length proteins are needed. This is especially important for human cellular proteins that may be tolerogenic in mice because of high homology with murine proteins. Strong immunogens presenting the target protein sequence on a suitable carrier may break the tolerance barrier and increase the immunogenicity of non-immunogenic proteins or protein segments.

In the current study, we designed novel recombinant immunogens based on pseudotype VLPs consisting of two HaPyV-derived capsid proteins—an intact VP1 protein and modified VP2 protein harbouring the target protein sequence at VP2 N terminus. As a target sequence, we have used full-length cellular protein of high diagnostic relevance p16^INK4A^ that is considered to be a potential marker for cells transformed by high-risk human papillomavirus (HPV). We have demonstrated that pseudotype VLPs consisting of an intact VP1 protein and VP2 protein fused with the p16^INK4A^ antigen at its N terminus induced a strong antibody response against the target sequence which allowed generation of p16^INK4A^-specific MAbs.

## 2. Materials and Methods

### 2.1. Production of Pseudotype VLPs Harbouring Full-Length p16^INK4A^ Protein

All DNA manipulations were carried out according to standard procedures [10]. Enzymes and kits for DNA manipulations were purchased from Thermo Scientific Fermentas (Vilnius, Lithuania). Recombinant plasmids were screened in *E.coli* DH10B cells. The synthetic gene encoding the full length p16^INK4A^ protein (synthesized by Integrated DNA Technologies, BVBA, Leuven, Belgium) was fused to hamster polyomavirus (HaPyV) VP2 gene modified at its N terminus in the plasmid pFGG3-VP1/VP2Bg. This plasmid was constructed by inserting HaPyV VP1 gene into GAL 7 expression cassette and modified HaPyV VP2 gene under GAL10-PYK1 hybrid promoter into yeast expression vector pFGG3 [11]. To construct the modified HaPyV VP2 gene, the sequence encoding 1–100 aa was deleted and GSS linker coding sequence and the BglII restriction site were introduced at its N terminus for a fusion with p16^INK4A^ coding sequence. The resulting plasmid pFGG3-VP1/VP2-p16 was used for the transformation of the *Saccharomyces cerevisiae *strain AH22-214 (a, leu2-3, 112, his4-519). Transformed yeast cells were grown in YEPD medium (yeast extract 1%, peptone 2%, and glucose 2%) supplemented with 5 mM formaldehyde at 30°C. The production of the recombinant protein was induced after 24 h of cultivation by adding galactose until 3% final concentration. After 18 h growth the yeast cells were harvested by centrifugation and stored at −20°C until use. The expression of recombinant VP1 and VP2 proteins was verified by gel electrophoresis and Western blot analysis of the yeast cell lysate as decribed hereinafter.

### 2.2. Purification and Electron Microscopy Analysis of Pseudotype VLPs

*S. cerevisiae* yeast biomass harbouring recombinant proteins was resuspended and homogenized in DB 450 buffer (450 mM NaCl, 1 mM CaCl_2_, 0.001% Trition X-100, 0.25 M L-Arginine in 10 mM Tris/HCl-buffer, pH 7.2) containing 2 mM phenylmethylsulfonyl fluoride (PMSF) and EDTA-free Complete Protease Inhibitor Cocktail (Thermo Scientific Fermentas) and mechanically disrupted using French press. After centrifugation, the supernatant was collected and loaded onto a 20–60% sucrose gradient. After centrifugation at 25,000 rpm (Rotor SW28, Beckman, USA) overnight the fractions of 0.5 mL were collected and subjected to SDS-PAGE. The fractions containing proteins of 42 and 45 kDa corresponding to VP1 and p16^INK4A^ fused with VP2 (VP2-p16^INK4A^), respectively, were pooled and diluted in buffer DB 150 (150 mM NaCl, 1 mM CaCl_2_ and 0.001% Trition X-100 in 10 mM Tris/HCl-buffer pH 7.2). The mixture was subjected to ultracentrifugation overnight at 100,000 ×g (Beckman) on CsCl gradient with densities from 1.23 to1.42 g/mL. The collected fractions were analyzed as described previously. As recombinant VP1 and VP2-p16^INK4A^ proteins were almost identical according to their molecular mass, the presence of VP2-p16^INK4A^ protein was verified by Western blot using in-house produced murine polyclonal antibodies against VP2 protein. Fractions containing VP1/VP2-p16^INK4A^ protein were diluted and precipitated by ultracentrifugation for 4 h, then dissolved in phosphate buffered saline (PBS), and dialyzed against PBS. The dialyzed VP1/VP2-p16^INK4A^ protein was aliquoted and lyophilized.

The VLP formation was verified by examination of the purified proteins by Morgagni 268 electron microscope (FEI Inc., Hillsboro, OR, USA). Protein samples were placed on 400-mesh carbon-coated palladium grids and negatively stained with 2% aqueous uranyl acetate.

### 2.3. Production of GST-Fused p16^INK4A^ Protein

To produce p16^INK4A^ protein fused to glutathione S-transferase (GST) in *E.coli*, the DNA sequence encoding p16^INK4A^ protein was cloned into the expression vector pGEX-5x (Amersham). The resulted plasmid pGEX-5x-p16 was used to transform *E.coli* strain BL1. The expression of GST-fused p16^INK4A^ protein was confirmed by SDS-PAGE and Western blot analysis with anti-GST antibodies (GE Healthcare, Uppsala, Sweden). The GST-p16^INK4A^-fused protein was purified using Glutathione Sepharose 4 Fast Flow (GE Healthcare Bio-Sciences AB SE-751 84) following the manufacturer's recommendations.

### 2.4. Immunization of Mice and Generation of Monoclonal Antibodies

BALB/c mice (obtained from a breeding colony at the Department of Immunology of the Center for Innovative Medicine, Vilnius, Lithuania) were immunized at days 0, 28, and 56 by a subcutaneous injection of 50 *μ*g of either recombinant pseudotype VLPs harbouring p16^INK4A^ protein or purified GST-p16^INK4A^-fused protein. For an initial immunization, the antigen was emulsified in complete Freund adjuvant (Sigma). Subsequent immunizations were performed without an adjuvant, with the antigen dissolved in PBS. Antisera were collected two weeks after the second injection and tested for the presence of antibodies specific to p16^INK4A^ protein. The mouse with the highest antibody titer against pseudotype VLPs was selected for the development of MAbs. Hybridomas were generated essentially as described by Kohler and Milstein [12]. Three days after the final injection, mouse spleen cells were fused with Sp2/0-Ag 14 mouse myeloma cells using polyethylene glycol 1500 (PEG/DMSO solution, HybriMax, Sigma). Hybrid cells were selected in growth medium supplemented with hypoxantine, aminopterin, and thymidine (50x HAT media supplement, Sigma-Aldrich, St. Louis, USA). Samples of supernatant from wells with viable clones were screened by an indirect enzyme-linked immunosorbent assay (ELISA) using recombinant VLPs and GST-fused p16^INK4A^ protein as described hereinafter. Hybridomas secreting specific antibodies to p16^INK4A^ protein were subcloned twice by a limiting dilution assay. Hybridoma cells were maintained in complete Dulbecco's modified Eagle's medium (DMEM, Biochrom) containing 15% fetal calf serum (Biochrom) and antibiotics. Antibodies in hybridoma culture supernatants were isotyped using the Mouse Monoclonal Antibody Isotyping kit (ISO-2, Sigma) in accordance with the manufacturer's protocol. All procedures involving experimental mice were performed under controlled laboratory conditions in strict accordance with the Lithuanian and European legislation.

### 2.5. SDS-PAGE

Proteins were analysed by electrophoresis on 12.5% sodium dodecylsulfate-polyacrylamide gels (SDS-PAGE) followed by Coomassie brilliant blue staining. The SDS-PAGE sample buffer (Thermo Scientific Fermentas) was added to the prepared protein samples, boiled for 5 min, applied to a polyacrylamide gel, and run in SDS-Tris-glycine buffer. Protein bands were visualized by staining with Coomassie brilliant blue (Sigma).

### 2.6. Western Blot Analysis

The proteins were separated by SDS-PAGE and electrotransferred to Immobilon P membrane (Millipore). The membranes were blocked with 5% milk in PBS for 2 h at room temperature (RT). The membranes were then incubated for 1 h at RT with primary antibodies at working dilution and subsequently incubated with goat antimouse IgG conjugated to horseradish peroxidase (HRP) (Bio-Rad) diluted 1 : 2000 in PBS with 0.1% Tween 20 (PBST). The enzymatic reaction was developed using tetramethylbenzidine (TMB) *ready-to-use* chromogenic substrate (Sigma). As primary antibodies for the identification of VP1/VP2-p16^INK4A^ proteins, mouse MAb against HaPyV VP1, clone 3D10 [9], and polyclonal antibodies produced *in-house *against HaPyV VP2 protein were used (dilution 1 : 1000 in PBST). For analysing MAb specificity, undiluted hybridoma supernatants were used.

### 2.7. Indirect ELISA

Polystyrene microtiter plates (Nerbe) were coated with 100 *μ*l/well of the antigen diluted in coating buffer (0.05 M sodium carbonate, pH 9.6) to a concentration of 5 *μ*g/mL by incubation overnight at 4°C. The coated plates were blocked with 150 *μ*L/well of 1% BSA for 2 h at RT. Plates were rinsed twice with PBST. Antiserum samples or hybridoma growth medium were diluted in PBST, added to the wells, and incubated for 1 h at RT. The plates were rinsed 3 times with PBST and incubated for 1 h with HRP-conjugated goat antimouse IgG (Bio-Rad) diluted 1 : 2000 in PBST. The plates were rinsed 5 times with PBST. The enzymatic reaction was visualized by the addition of 100 *μ*L of ready-to-use TMB substrate (Sigma) to each well. After 10 min of incubation at RT, the reaction was stopped by adding 50 *μ*L/well of 10% sulphuric acid. The optical density (OD) was measured at 450 nm (reference filter 620 nm) in a microplate reader (Tecan, Groedig, Austria).

### 2.8. Immunohistochemistry Analysis

Immunohistochemical staining was performed on paraffin-embedded samples of cervical squamous cell carcinomas and nonneoplastic cervical tissue selected from archival materials at the National Specialized Hospital for Active Treatment in Oncology, Sofia, Bulgaria. Haematoxylin and eosin-stained slides of all samples were reviewed by a pathologist and their histopathological diagnoses were reconfirmed. Sections (approximately 5 *μ*m thick) were cut and mounted on poly-l-lysine coated microscope slides (Thermo Scientific). Samples were deparaffinized in xylene and rehydrated in graded alcohols. Antigen retrieval was performed in 0.01 M citrate buffer (pH 6.0) in a heating bath for 20 min at 97°C. Endogenous peroxidase activity was blocked by incubating the sections in 3% H_2_O_2_ for 5 min. After blocking the nonspecific binding with 3% BSA in PBS for 3 h at RT, slides were incubated with the primary antibody (mouse polyclonal antibody raised against pseudotype VLPs harbouring p16^INK4A^ protein; 1 : 300, or mouse polyclonal antibody raised against GST-p16^INK4A^ fused protein; 1 : 100) and left overnight in moist chambers at 4°C. The bound antibody was visualized using a biotinylated secondary antibody, peroxidase-labelled streptavidin, and DAB substrate-chromogen (LSAB2 System-HRP, Dako, Denmark) according to the manufacturer's protocol. Sections were counterstained in hematoxylin, mounted and analyzed under light microscopy. As a negative control, irrelevant mouse polyclonal antibody raised against yeast-expressed hPIV3 nucleocapsid protein was used [13].

### 2.9. Flow-Cytometry

Adherent human cervical epithelial HeLa cells (ATCC Cat. No. CCL-2) were cultivated in RPMI-1640 growth medium (Biochrom, Berlin, Germany) supplemented with 10% fetal bovine serum (Biochrom) and antibiotics. The cells were grown at 37°C and 5% CO_2_ to approximately 70% confluence, harvested, resuspended in Fixation/Permeabilization solution (BD Biosciences, Franklin Lakes, USA), and incubated for 20 minutes at 4°C. The cells were washed two times with BD Perm/Wash buffer (BD Biosciences) and transferred to plastic tubes for immunofluorescent staining, 10^6^ cells per test. One hundred *μ*L of BD Perm/Wash buffer containing 5 *μ*g/mL of the MAb against p16^INK4A^ or appropriate positive and negative controls were added to the cells and incubated at 4°C for 30 min. As a positive control, commercial anti-CDK2A/p16^INK4A^ MAb, clone DCS-50.1/H4 (Abcam, Cambridge, UK) was used (10 *μ*g/mL). As a negative control, irrelevant MAb of IgG1 isotype against yeast-expressed hPIV3 nucleocapsid protein was used (10 *μ*g/mL) [13]. After incubation, the cells were washed two times with BD Perm/Wash buffer and then incubated for 30 min at 4°C in the dark with 50 *μ*L of BD Perm/Wash buffer containing a predetermined optimal concentration of FITC-conjugated goat antimouse IgG (BD Pharmingen, Franklin Lakes, USA). Finally, the cells were washed two times with BD Perm/Wash buffer and resuspended in Staining Buffer (BD Pharmingen) prior to flow cytometric analysis. Cells were analyzed with CyFlow^R^space flow cytometer (Partec, Muenster, Germany). Not less than 20.000 events per test were evaluated with FloMax 2.7 software.

## 3. Results

**Figure 1:**
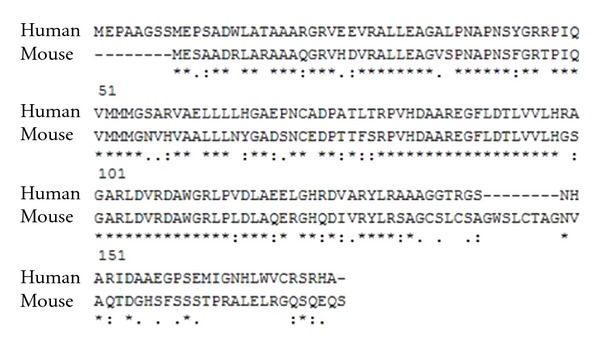
Alignment of aa sequences of human and murine p16^INK4A^ using ClustalLW computer program. Identical and similar aa residues are marked by asterisks and dots, respectively.

Full-length human p16^INK4A^ protein (16 kDa, 133 aa-long) was selected as a target protein for the generation of pseudotype VLPs and further immunization experiments. The alignment of aa sequences of human and murine p16^INK4A^ proteins using ClustalLW and BLAST computer programs revealed 75% sequence homology (Figure 1). High number of identical and similar aa residues indicated the low immunogenicity of human p16^INK4A^ in mice; therefore, the antigen was considered to be suitable as a target sequence for presenting on pseudotype VLPs.

**Figure 2:**
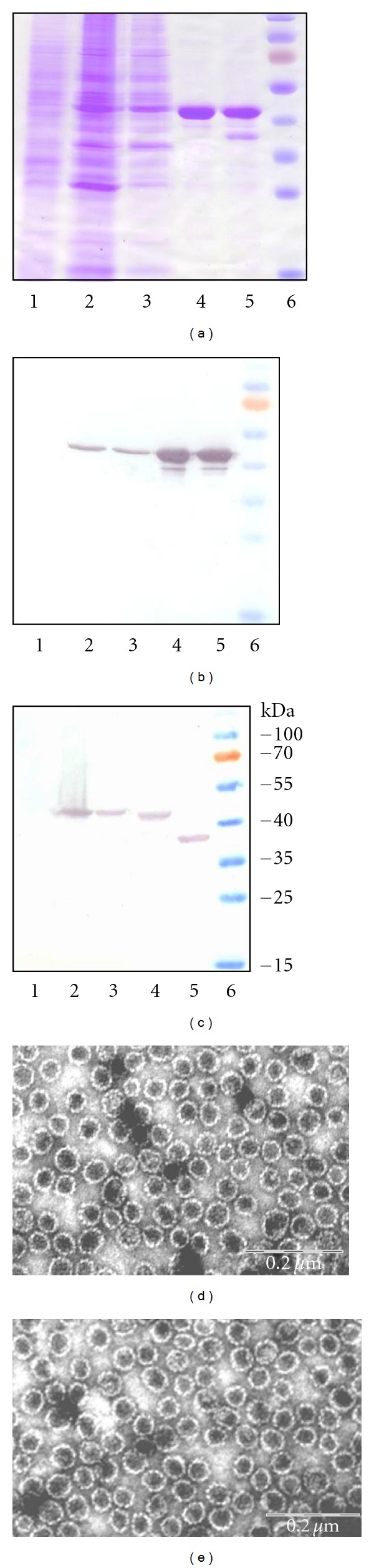
Analysis of the expression of VP1/VP2-p16^INK4A^ pseudotype VLPs in yeast. (a) Coomassie blue-stained SDS-PAGE. (b) Western blot with the MAb 3D10 against VP1 protein. (c) Western blot with polyclonal antibody against VP2 protein. The same samples were run on each gel. In lanes, there are (1) negative control sample from crude lysate of *S. cerevisiae* cells transformed with the empty vector pFGG3, (2) crude lysate of yeast transformed with pFGG3-VP1/VP2-p16 plasmid, (3) the soluble fraction recovered after centrifugation of crude lysate of yeast transformed with pFGG3-VP1/VP2-p16 plasmid, (4) VLPs consisting of VP1 protein and fusion protein VP2-p16 purified using sucrose and CsCl gradients, (5) VLPs consisting of VP1 protein and nonmodified VP2 protein purified using sucrose and CsCl gradients, and (6) prestained protein molecular mass marker (Thermo Scientific Fermentas). (d and e) Electron microscopy pictures of VP1/VP2-p16^INK4A^ (d) and nonmodified VP1/VP2 (e) pseudotype VLPs, stained with 2% aqueous uranyl acetate solution and examined by Morgagni 268 electron microscope.

For the construction of expression plasmids, synthetic gene encoding full-length p16^INK4A^ sequence was inserted into yeast expression vector pFGG3-VP1/VP2Bg designed for the coexpression HaPyV VP1 protein together with VP2 protein truncated until the 101 aa residue. The resulted plasmid pFGG3-VP1/VP2-p16 was used to transform yeast *S.cerevisiae* strain AH22-214. The SDS-PAGE analysis of crude lysates of transformed yeast cells revealed an overlapping protein band of approximately 42–45 kD because the molecular mass of full-length VP1 protein and VP2- p16^INK4A^ fused protein was very similar (42 and 45 kD, resp.) (Figure 2(a), lane 2). The corresponding protein band was not visible in the lysate of yeast cells transformed with empty vector pFGG3 used as a negative control (Figure 2(a), lane 1). Protein bands representing the VP1 protein and VP2-p16^INK4A^ fused protein were specifically immunostained with the respective antibodies against HaPyV VP1 and VP2 proteins (Figures 2(b) and 2(c), lane 2). The soluble fraction of the lysate of transformed yeast cells was subjected to ultracentrifugation in sucrose and CsCl density gradients. The purified recombinant VP1/VP2-p16^INK4A^ proteins were analyzed by SDS PAGE and Western blot. According to SDS-PAGE data, the purity of VP1/VP2-p16^INK4A^ proteins after the ultracentrifugation step was about 99% (Figure 2(a), lane 4). The identity of purified proteins was confirmed by Western blot analysis using specific antibodies (Figures 2(b) and 2(c), lane 4). Electron microscopy analysis of the purified negatively stained VP1/VP2-p16^INK4A^ proteins confirmed the formation of VLPs of about 45 nm in diameter (Figure 2(d)) similar in their size and shape to the nonmodified VP1/VP2 VLPs (Figure 2(e)) and to native viral capsids.

The pseudotype VLPs were used to immunize BALB/c mice to generate antibodies against the inserted target sequence. In parallel, the BALB/c mice were immunized with purified GST-fused p16^INK4A^ protein. After 2 immunizations, the titers of antibodies specific to VP1/VP2-p16^INK4A^ determined by an indirect ELISA in the sera of mice immunized with pseudotype VLPs ranged from 1 : 16000 to 1 : 32000 (data not shown). To confirm the specificity of the antisera with p16^INK4A^ protein, their specificity was analyzed by Western blot using fused protein GST-p16^INK4A^ expressed in *E.coli. *Specific immunostaining of GST-p16^INK4A^ fuse was observed, which confirms that the antibodies raised against pseudotype VLPs recognize the p16^INK4A^ sequence (data not shown). In contrast, the antisera raised against GST-p16^INK4A^ fused protein recognized only the antigen used for immunization (titers after 2 immunizations ranged 1 : 4000–1 : 12000) and did not show any reactivity with the p16^INK4A^ sequence displayed on VLPs (data not shown).

**Figure 3:**
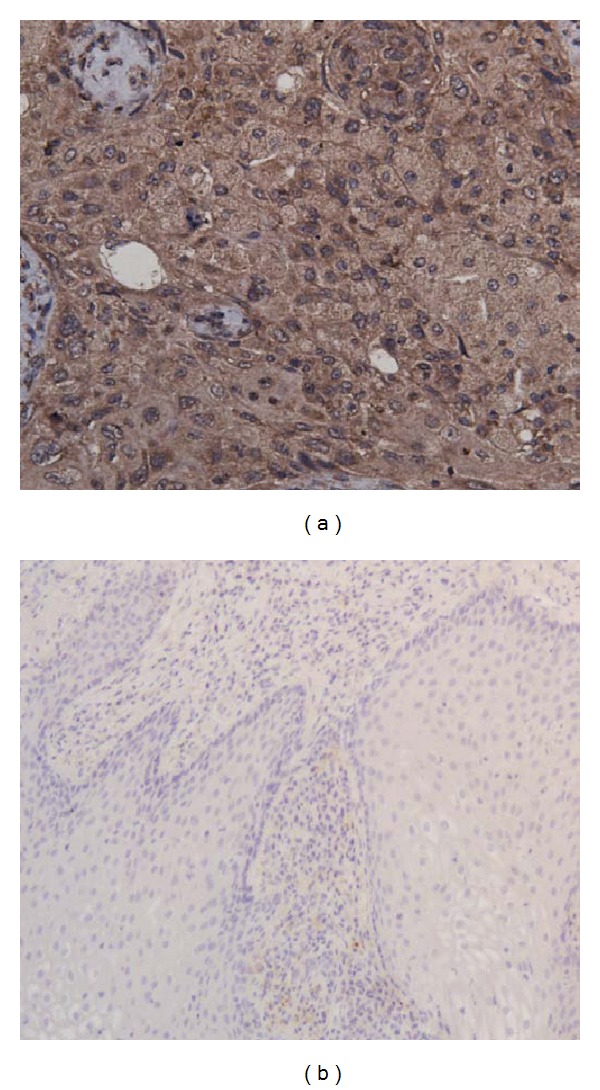
Immunohistochemical analysis of cervical tissues using mouse polyclonal antibody raised against pseudotype VLPs harbouring the p16^INK4A^ protein. (a) Invasive squamous cell carcinoma; (b) nonneoplastic cervical tissue (condyloma).

To investigate the reactivity of the antisera with cellular p16^INK4A^ protein, they were applied to the immunohistochemistry analysis (IHC) of cervical tissue specimens. The antisera raised against pseudotype VLPs showed specific immunostaining of malignant cervical tissue containing HPV-transformed cells and did not react with nonneoplastic cervical tissue (Figure 3). This demonstrates the reactivity of the antisera raised against pseudotype VLPs with the cellular p16^INK4A^ protein present in malignant cervical tissue. In contrast, the antisera raised against GST-fused p16^INK4A^ protein did not show any reactivity in IHC (data not shown). Therefore, no further experiments with mice immunized with GST-p16^INK4A^ fused protein were performed.

**Figure 4:**
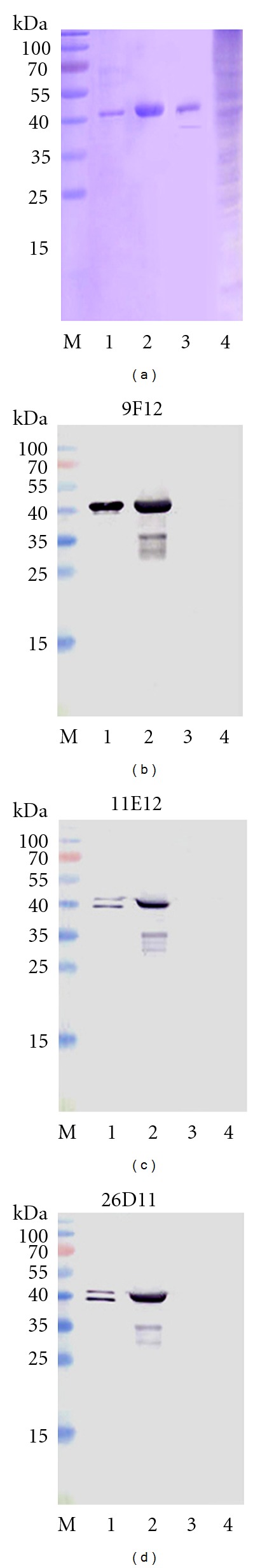
The reactivity of the MAbs raised against VP1/VP2-p16^INK4A^ pseudotype VLPs with the p16^INK4A^ protein sequence in Western blot. (a) Coomassie blue-stained SDS-PAGE. (b–d) Western blot with the MAbs raised against pseudotype VLPs, clones 9F12 (b), 11E12 (c), and 26D11 (d). Lane M: prestained protein molecular mass marker (Thermo Scientific Fermentas); lane 1: purified GST-fused p16^INK4A^ protein; lane 2: purified pseudotype VP1/VP2-p16^INK4A^ VLPs; lane 3: purified nonmodified VP1/VP2 VLPs; lane 4: crude lysate of nontransformed *S. cerevisiae* cells.

**Figure 5:**
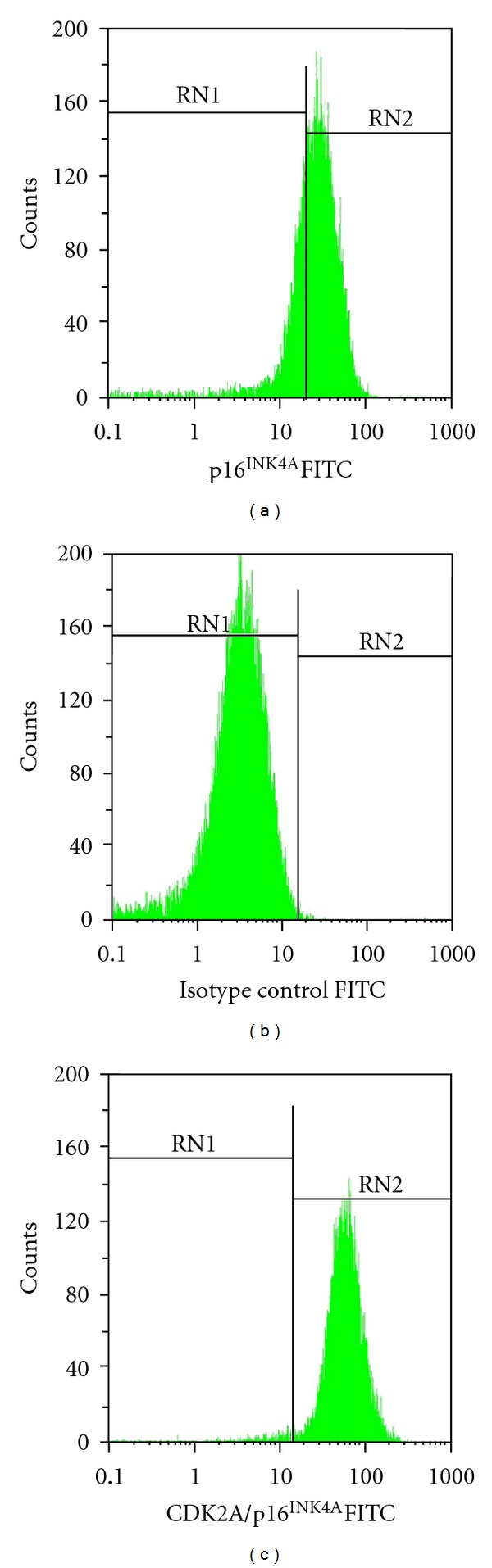
Flow-cytometry analysis of HeLa cells immunostained with the MAbs: (a) clone 9F12 (IgG1 subtype) raised against pseudotype VLPs harbouring the p16^INK4A^ protein; (b) negative control, irrelevant Mab of IgG1 subtype; (c) positive control, commercial MAb against p16INK4A protein (Abcam).

 Spleen cells of mice immunized with pseudotype VLPs were used to generate the MAbs against p16^INK4A^ protein. Three stable hybridoma cell lines producing p16^INK4A^-specific MAbs of IgG isotype (IgG1 subtype) were generated. The MAbs reacted specifically in Western blot both with pseudotype VP1/VP2-p16^INK4A^ VLPs and GST-p16^INK4A^ fuse but did not react either with yeast cell lysate or nonmodified VP1/VP2 VLPs used as a negative control (Figure 4). To prove the reactivity of the MAbs with native intracellular p16^INK4A^ protein, they were applied to flow-cytometry analysis of HeLa cells that represent HPV-18–transformed cervical epithelial cells. The intracellular staining of HeLa cells confirmed the ability of the MAbs to recognize the intracellular native p16^INK4A^ and revealed about 65% of p16^INK4A^-positive cells (Figure 5(a)). The positivity of HeLa cells for p16^INK4A^-protein was confirmed by their specific immunostaining with the commercial MAb against p16^INK4A^ protein (Figure 5(b)). No specific immunostaining of HeLa cells was observed with an irrelevant MAb of the same isotype (Figure 5(c)). Thus, polyclonal and monoclonal antibodies raised against pseudotype VLPs harbouring the full-length p16^INK4A^ sequence were reactive with cellular native p16^INK4A^ protein. This is an indirect evidence that the p16^INK4A^ molecule displayed on the surface of pseudotype VLPs is natively folded.

In conclusion, our results demonstrate that pseudotype VLPs represent highly efficient carrier for cellular antigens and elicit a strong antibody response against the target protein presented on the surface of VLPs.

## 4. Discussion

The aim of the current study was to design novel recombinant antigen capable to display surface-exposed foreign protein sequences and enhance their immunogenicity. Such recombinant antigens may be applied for the generation of antibodies against cellular proteins of low immunogenicity. As a target protein for the construction of the recombinant antigen, we have selected the cellular marker p16^INK4A^ that represents an indirect indicator of cell cycle dysregulation associated with high-risk HPV infection. The expression of p16^INK4A^ is specifically induced in HPV-infected cells by HPV E7 protein that inactivates regulatory protein pRb (*retinoblastoma gene product*) and upregulates transcription factor E2F, which allows the *cdk* gene transcription. The product of *cdk* gene is protein p16^INK4A^. Several studies examined the p16^INK4A^ protein by immunocytochemical analysis and confirmed its diagnostic relevance as a biomarker for dysplastic squamous and glandular cells of the cervix [14–16].

Based on the alignment of aa sequences of human and murine p16^INK4A^ proteins that revealed high degree of homology, the low immunogenicity of human p16^INK4A^ in mice was predicted. To enhance its immunogenicity, we have constructed pseudotype VLPs consisting of an intact HaPyV VP1 protein and VP2 protein fused with the target antigen—p16^INK4A^ protein—at VP2 N terminus. Both recombinant proteins coexpressed in yeast *S.cerevisiae* were able to self-assemble to pseudotype VLPs harbouring the inserted target antigen. The shape and size of the pseudotype VLPs was similar to that observed for the native HaPyV capsids [17]. From the resolved crystal structures of the virions of SV-40 [18] and murine polyomavirus [19] it is known that the capsid of polyomaviruses mainly consists of 72 pentamers formed by 360 copies of the VP1 protein. One minor capsid protein, either VP2 or VP3, binds in the central 5-fold cavity of each VP1 pentamer [20]. The C terminal part of VP2 protein is necessary for it interaction with VP1 pentamer [20]; therefore, we have used the VP2 N-terminus to join the full-length p16^INK4A^ molecule. The ratio of VP1 : VP2 proteins in HaPyV capsid is 360 : 72 [20]; consequently up to 72 chimeric VP2-p16 molecules might be incorporated into one VLP. It was supposed that the inserted p16^INK4A^ sequence will be displayed on the surface of pseudotype VLPs thus allowing its accessibility to the B cells. The immunogenicity of pseudotype VLPs in mice was proven by immunization experiments and subsequent generation of p16^INK4A^-specific MAbs. Polyclonal antibodies raised against pseudotype VLPs specifically recognized p16^INK4A^ sequence both by Western blot using recombinant *E.coli*-expressed GST-p16^INK4A^ fuse and by the immunohistochemistry using cervical tissue specimens. In contrast, the immunization of mice with the same doses of GST-fused p16^INK4A^ protein did not induce formation of p16^INK4A^-specific antibodies, most likely due to the masking of the target sequence by the GST. The immunization of mice with pseudotype VLPs allowed generation of several MAbs that specifically recognized recombinant p16^INK4A^ protein by both ELISA and Western blot. Moreover, the reactivity of the MAbs with the intracellular native p16^INK4A^ was demonstrated. The ability of the MAbs to recognize native cellular p16^INK4A^ suggests proper folding of the target sequence displayed on the surface of pseudotype VLPs. The obtained results demonstrate the efficiency of pseudotype VLPs in presenting foreign protein sequences to B cells for the generation of target-specific antibodies.

## 5. Conclusions

Hamster polyomavirus-derived proteins VP1 and VP2 capable to self-assemble to VLPs can be employed to construct chimeric proteins with improved immunogenicity. Yeast-expressed pseudotype VLPs consisting of the intact VP1 protein and VP2 protein fused to the cellular antigen p16^INK4A^ are immunogenic and induce antibodies specific to the p16^INK4A^ sequence. The current study demonstrates the potential of pseudotype VLPs with inserted target antigen as a new type of immunogens to generate antibodies of high diagnostic relevance.

##  Conflict of Interests

The authors declare that there are no competing interests.
